# Functional characterization of cannabidiol effect on the serotonergic neurons of the dorsal raphe nucleus in rat brain slices

**DOI:** 10.3389/fphar.2022.956886

**Published:** 2022-09-06

**Authors:** Aitziber Mendiguren, Erik Aostri, Elena Alberdi, Alberto Pérez-Samartín, Joseba Pineda

**Affiliations:** ^1^ Department of Pharmacology, Faculty of Medicine and Nursing, University of the Basque Country (UPV/EHU), Leioa, Spain; ^2^ Achucarro Basque Center for Neuroscience, Department of Neuroscience, Faculty of Medicine and Nursing, University of the Basque Country (UPV/EHU), Leioa, Spain

**Keywords:** cannabidiol, 5-HT_1A_ receptor, dorsal raphe nucleus, slice, firing, rat, calcium

## Abstract

Cannabidiol (CBD), the main non-psychoactive cannabinoid found in the cannabis plant, elicits several pharmacological effects *via* the 5-HT_1A_ receptor. The dorsal raphe nucleus (DRN) is the main serotonergic cluster in the brain that expresses the 5-HT_1A_ receptor. To date, the effect of CBD on the neuronal activity of DRN 5-HT cells and its interaction with somatodendritic 5-HT_1A_ autoreceptors have not been characterized. Our aim was to study the effect of CBD on the firing activity of DRN 5-HT cells and the 5-HT_1A_ autoreceptor activation by electrophysiological and calcium imaging techniques in male Sprague–Dawley rat brain slices. Perfusion with CBD (30 μM, 10 min) did not significantly change the firing rate of DRN 5-HT cells or the inhibitory effect of 5-HT (50–100 μM, 1 min). However, in the presence of CBD (30 μM, 10 min), the inhibitory effects of 8-OH-DPAT (10 nM) and ipsapirone (100 nM) were reduced by 66% and 53%, respectively. CBD failed to reverse ipsapirone-induced inhibition, whereas perfusion with the 5-HT_1A_ receptor antagonist WAY100635 (30 nM) completely restored by 97.05 ± 14.63% the firing activity of 5-HT cells. Administration of AM251 (1 µM), MDL100907 (30 nM), or picrotoxin (20 μM) did not change the blockade produced by CBD (30 μM) on ipsapirone-induced inhibition. Our study also shows that CBD failed to modify the KCl (15 mM, 4 min)-evoked increase in [Ca^2+^]_i_ or the inhibitory effect of ipsapirone (1 μM, 4 min) on KCl-evoked [Ca^2+^]_i_. In conclusion, CBD does not activate 5-HT_1A_ autoreceptors, but it hindered the inhibitory effect produced by selective 5-HT_1A_ receptor agonists on the firing activity of DRN 5-HT cells through a mechanism that does not involve CB_1_, 5-HT_2A,_ or GABA_A_ receptors. Our data support a negative allosteric modulation of DRN somatodendritic 5-HT_1A_ receptor by CBD.

## Introduction

∆^9^-tetrahydrocannabinol (∆^9^-THC) and cannabidiol (CBD) are the main cannabinoids present in the plant *Cannabis sativa*. ∆^9^-THC binds to the cannabinoid CB_1_ receptor to elicit psychotomimetic effects, while CBD exhibits low affinity for the CB_1_ receptor and lacks psychoactive effects (Pisanti et al., 2017; Britch et al., 2021). CBD has been shown to target, among others, the G_i_/_o_ protein-coupled 5-HT_1A_ receptor, the Gαq protein-coupled 5-HT_2A_ receptor, and the GABA_A_ receptor (Russo et al., 2005; Bakas et al., 2017; De Almeida and Devi, 2020; Britch et al., 2021).

The dorsal raphe nucleus (DRN) is the main serotonergic (5-HT) nucleus in the rat brain, which expresses G_i/o_ protein-coupled 5-HT_1A_ somatodendritic receptors. Stimulation of 5-HT_1A_ autoreceptor of DRN 5-HT cells by 5-HT_1A_ receptor agonists activates G protein–coupled inwardly rectifying potassium channels (GIRKs), and inhibits voltage-gated Ca^+2^ channels (VGCCs), leading to a reduction of the neuronal activity and a decrease of 5-HT release in the DRN and its projection areas (Andrade et al., 2015; Courtney and Ford, 2016). The activity of 5-HT cells in the DRN *in vitro* is also regulated by non-5-HT cells, primarily GABAergic interneurons (Celada et al., 2001; Challis et al., 2013; Hernández-Vázquez et al., 2019), which have been shown to be inhibited by GABA_A_ receptor and activated by 5-HT_2A_ receptors (Liu et al., 2000).

The DRN is involved in the regulation of different physiological functions and pathological states including mood, sleep–wake cycle, arousal, anxiety, and control of pain (Lowry et al., 2008; Monti, 2010; McDevitt and Neumaier, 2011; Campion et al., 2016). Thus, stimulation of DRN 5-HT_1A_ autoreceptors induces anxiolytic effects (Garcia-Garcia et al., 2014), and desensitization of 5-HT_1A_ autoreceptors has been linked to clinical effects of antidepressants (Piñeyro and Blier, 1999; Garcia-Garcia et al., 2014). Several studies have demonstrated that classical cannabinoids regulate DRN neurons (Mendiguren et al., 2018). CB_1_ receptor agonists and antagonists change the firing rate of DRN 5-HT cells *in vivo* and *in vitro* (Gobbi et al., 2005; Bambico et al., 2007; Mendiguren and Pineda, 2009). Furthermore, numerous behavioral effects produced by cannabinoids have been suggested to occur through their action on the 5-HT system/DRN such as anxiolytic (Braida et al., 2007) or antidepressant effects (Gobbi et al., 2005).

Over the last years, research has focused on studying the pharmacological effects of the non-psychoactive cannabinoid CBD (Pisanti et al., 2017; Britch et al., 2021). It has been shown that CBD elicits, *via* 5-HT_1A_ receptor, antiepileptic (Silvestro et al., 2020), anticataleptic (Gomes et al., 2013), neuroprotective (Fernández-Ruiz et al., 2013; Silvestro et al., 2020), antiemetic (Rock et al., 2012; Bolognini et al., 2013), anxiolytic (Resstel et al., 2009; Marinho et al., 2015; De Gregorio et al., 2019), antidepressant (Zanelati et al., 2010; Linge et al., 2016; Sartim et al., 2016), antipsychotic (Rodrigues da Silva et al., 2020), or analgesic effects (De Gregorio et al., 2019; Jesus et al., 2019; Britch et al., 2021). Furthermore, CBD increased [(35) S]GTPγS binding, reduced cAMP, and displaced [3H]8-OH-DPAT from cloned human 5-HT_1A_ receptors, suggesting a partial agonism at 5-HT_1A_ receptors (Russo et al., 2005). It has also been shown that CBD enhances the ability of 8-OH-DPAT to stimulate [(35) S] GTPγS binding in rat brainstem membranes *in vitro* (Rock et al., 2012). However, the brain nuclei involved in the pharmacological effects of CBD remain to be studied. To date, there is only one electrophysiological study in which the effect of systemically applied CBD on the firing rate of DRN 5-HT cells was studied (De Gregorio et al., 2019). Thus, the effect of CBD on the activity of 5-HT cells and its interaction with somatodendritic 5-HT_1A_ autoreceptors in slices from the DRN have not been characterized yet. Our aim was to study the effect of CBD on the neuronal activity of DRN 5-HT cells and on the 5-HT_1A_ autoreceptor activation by electrophysiological and calcium imaging techniques in rat brain slices.

## Materials and methods

### Brain slice preparation

Experiments were performed in male Sprague–Dawley (200–300 g) rats housed under standard laboratory conditions (22°C, 12 h light/dark cycles) with free access to food and water. All the experiments were carried out according to EU Directive 2010/63 on the protection of animals used for scientific purposes and approved by the local Ethical Committee for Research and Teaching of the University of the Basque Country (UPV/EHU, Spain) and the Department of Sustainability and Natural Environment of Provincial Council from Bizkaia (ref. CEEA M20-2018-025). All the efforts were made to minimize animal suffering and reduce the number of animals used.

Animals were anesthetized with chloral hydrate (400 mg kg^−1^ i. p.) and then decapitated. The brain was rapidly excised after death and placed in ice-cold artificial cerebrospinal fluid (ACSF), where NaCl was substituted by sucrose to improve neuronal viability. Coronal brainstem sections of 600 µm thickness containing the DRN were cut using a vibratome and incubated at 33°C in a modified Haas-type interface chamber continuously perfused with ACSF at a flow rate of 1.5 ml min^−1^ (Mendiguren and Pineda, 2009). A recovery period of 2 h was allowed before starting electrophysiological recordings. For calcium imaging experiments, coronal slices of 300 µm thickness containing the DRN were cut. The ACSF used for both electrophysiological and calcium imaging experiments contained (in mM) NaCl 130, KCl 3, NaH_2_PO_4_ 1.25, MgSO_4_ 2, CaCl_2_ 2, NaHCO_3_ 20, and d-glucose 10 and was equilibrated with 95% O_2_ plus 5% CO_2_ (pH = 7.34).

### Extracellular recordings

Single-unit extracellular recordings of DRN 5-HT neurons were made as previously described (Mendiguren and Pineda, 2009). The recording electrode, which consisted of an Omegadot glass micropipette, was pulled and filled with NaCl (0.05 M). The tip was broken back to a size of 2–5 µm (3–5 MΩ). The electrode was positioned in the DRN, which was identified visually as a dark area in the ventromedial part of the periaqueductal gray. The cells were recorded in the ventro-central region of the nucleus, which has been reported to mainly contain 5-HT neurons (Peyron et al., 2018). The extracellular signal from the electrode was passed through a high-input impedance amplifier and monitored on an oscilloscope and also with an audio unit. Individual neuronal spikes were isolated from the background noise with a window discriminator, and the firing rate was analyzed by means of a PC-based custom-made program, which generated histogram bars representing the cumulative number of spikes in consecutive 10-s bins (HFCP^®^, Cibertec SA, Madrid, Spain).

As the majority of 5-HT neurons in slice preparations are silent, due to an interruption of the noradrenergic excitatory input, the *α*_1_ adrenoceptor agonist phenylephrine (PE, 15 μM) was perfused from the beginning of each experiment to drive the firing activity as previously described (Mendiguren and Pineda, 2009). Only one cell was recorded in each slice and rat. DRN 5-HT cells were identified by their electrophysiological features: a regular discharging pattern, a slow firing rate, and a long-lasting biphasic positive-negative waveform (2 ms). Moreover, pharmacological criteria such as the inhibitory response to bath application of 5-HT (50–100 μM, 1 min) at the beginning of the experiment were used to confirm the identity of 5-HT cells (Aghajanian and Lakoski, 1984 Hajós and Sharp, 1996 Mendiguren and Pineda, 2009). We only considered as 5-HT neurons those cells that fulfilled both electrophysiological and pharmacological criteria.

### Ca^2+^ imaging experiments

Calcium influx was estimated by the 340/380 ratio method as described previously (Grynkiewicz et al., 1985). Cells were exposed to ACSF containing a lower concentration of NaCl equiosmotically substituted for a high concentration of KCl (15 mM) to evoke [Ca^2+^]i increase (Cheng et al., 1998). Ca^2+^ levels in the DRN were monitored by fluorescence microscopy using the Ca^2+^ indicator Fura-2-AM (Molecular Probes, Invitrogen, Barcelona, Spain). Slices were incubated with Fura-2-AM (5 μM and 1% of pluronic acid) for 50 min at 37°C. Experiments were performed in a chamber continuously perfused with incubation buffer at 1 ml/min. Drugs were perfused by a pump with a quartz/polyimide tube of 200 µm ID, attached to a perfusion chamber. The perfusion chamber was mounted on the stage of a Leica DMLFSA upright microscope equipped with a 150-W xenon lamp Polychrome V (T.I.L.L. Photonics, Martinsried, Germany) and an HCX Apo ×40water immersion objective (Leica). Cells were visualized with a CCD camera (EM CCD 9100; Hamamatsu Photonics Iberica, Barcelona, Spain). Several cells were recorded from each slice, and a maximum of two slices were taken from each rat. Overall, 11 rats were used, from which 12 slices were taken.

### Experimental design

To study the effect of CBD on the PE-driven firing activity of DRN 5-HT cells, we first perfused the vehicle of the drug (DMSO 0.08%, 10 min) and then the cannabinoid (30 μM, 10 min). To assess the effect of CBD (30 µM) on 5-HT_1A_ receptor-mediated inhibition of the firing rate, the endogenous ligand 5-HT (50–100 μM, 1 min), or the more selective 5-HT_1A_ receptor agonists 8-OH-DPAT (10 nM, 10 min) and ipsapirone (100 nM, 10 min) were applied in the absence and presence of the cannabinoid. The 5-HT_1A_ receptor agonists 8-OH-DPAT and ipsapirone show similar affinity for the 5-HT_1A_ receptor, but ipsapirone has been clinically studied. Therefore, to study the putative mechanisms involved in the effect of CBD on 5-HT_1A_ receptor activation, ipsapirone (100 nM, 10 min) was used as a 5-HT_1A_ receptor agonist and the CB_1_ receptor antagonist AM251 (1 µM), and the GABA_A_ receptor antagonist picrotoxin (20 μM) and the 5-HT_2A_ receptor antagonist MDL100907 (30 nM) were applied in the absence and presence of the cannabinoid. All the antagonists were perfused at least for 10 min before CBD administration. MDL100907 and picrotoxin were used to block the putative action of CBD at the 5-HT_2A_ receptor onto GABAergic interneurons and the subsequent inhibition produced by GABA, *via* GABA_A_ receptor, on 5-HT cells, respectively. AM251 was used to antagonize CB_1_ receptors mainly located at GABA interneurons in the DRN, which could also be targeted by CBD. In addition, AM251 would also block the putative effect of CBD on the CB_1_ receptor located at 5-HT or glutamatergic neurons. To determine if CBD (30 μM) behaved as a 5-HT_1A_ receptor antagonist, the cannabinoid was applied once the 5-HT cell had been completely inhibited with ipsapirone (100 nM, 10 min). After perfusion with the cannabinoid, the selective 5-HT_1A_ receptor antagonist WAY100635 (30 nM) was applied to restore the firing activity.

For calcium imaging experiments, [Ca^2+^]i increase was evoked by depolarization with KCl (15 mM, 4 min) due to the presence of low resting levels of [Ca ^2+^]i in slice preparations from the DRN. We studied KCl-evoked increase in [Ca^2+^]i in the presence of ipsapirone (1 µM), CBD (30 µM), or both. All drugs were applied for 2 min before evoking [Ca^2+^]i increase with KCl (15 mM) and also during KCl administration.

### Data analysis and statistical procedures

In electrophysiological assays, the firing rate of DRN 5-HT cells was recorded before, during, and after drug application throughout the experiment. The effect of CBD on PE-driven firing activity was calculated by subtracting the firing rate before CBD administration from the firing rate value at the time of the peak change. Then, this value was used to calculate the percentage change from the firing rate before CBD administration. To analyze the effect of CBD on the inhibitory effect of 5-HT_1A_ receptor agonists, we calculated the inhibition induced by 5-HT, 8-OH-DPAT, or ipsapirone in each cell in the absence and presence of CBD. The effect induced by 5-HT_1A_ receptor agonists was calculated as follows: E = FRpost-FRbasal, where FR basal is the mean firing rate (spikes/10 s) for a 60-s period immediately before 5-HT_1A_ receptor agonist application and FR post is the maximum change achieved during drug administration (spikes/10 s).

In calcium imaging experiments, image acquisition and data analysis were performed by the AquaCosmos software program (Hamamatsu Photonics Iberica). The area under the curve (AUC) for KCl-evoked [Ca^2+^] peak was measured for each stimulus. We first characterized the KCl-evoked effect in the absence of drugs by depolarizing DRN 5-HT cells with KCl twice. Then, the percentage from the first AUC for KCl-evoked [Ca^2+^] peak was calculated, and this value was used as a control for comparisons with the percentage of KCl-evoked increase in [Ca^2+^]i in the presence of ipsapirone or CBD. The effect of CBD on ipsapirone-induced inhibition of KCl-evoked [Ca^2+^]i increase was studied by comparing the percentage from the first AUC in the presence of CBD with that after perfusion with CBD and ipsapirone.

Data were given as mean ± standard error of the mean (SEM). Data before and after drug perfusion were compared by the paired Student’s *t* test. Comparisons between data from two independent groups were carried out by a two-sample (unpaired) Student’s *t* test and for more than two groups by two-way ANOVA followed by Bonferroni´s multiple comparison test. The computer programs GraphPad Prism (GraphPad Software, San Diego, California United States) and SPSS for windows were used for statistical evaluation. The level of significance was considered when *p* = 0.05.

### Drugs

(-) Cannabidiol (CBD), R-(+)-α-(2,3-dimethoxyphenyl)-1-[2-(4-fluorophenyl)ethyl]-4-piperinemethanol (MDL100907), N-(piperidin-1-yl)-5-(4-iodophenyl)-1-(2,4-dichlorophenyl)-4-methyl-1H-pyrazole-3-carboxamide (AM251), (2-[4-[4-(2-pyrimidinyl)-1-piperazinyl]butyl]-1,2-benzisothiazol-3(2H)-one-1,1-dioxide (ipsapirone), and phenylephrine (PE) hydrochloride were purchased from Tocris (Bristol, United Kingdom). 5-hydroxytryptamine (5-HT), (±)-8-hydroxy-2-(di-n-propylamino) tetralin] hydrobromide (8-OH-DPAT), N-[2-[4-(2-methoxyphenyl)-1-piperazinyl]ethyl]-N-2-pyridinylcyclohexanecarboxamide (WAY100635), and picrotoxin were purchased from Sigma (St Louis, MO, United States).

Stock solutions of AM251, MDL100907, ipsapirone, and CBD were prepared in dimethylsulfoxide (DMSO) and those of 5-HT, WAY100635, and 8-OH-DPAT in distilled water. Stock solutions were diluted in ACSF to their final concentration, just before each application. Picrotoxin and PE were directly dissolved in ACSF. Control assays were carried out with the vehicles at their maximal concentrations. The maximal final concentration of DMSO in the perfusion fluid was 0.09%.

## Results

### Effect of CBD on the firing rate of DRN 5-HT cells and on 5-HT-induced inhibition

**FIGURE 1:**
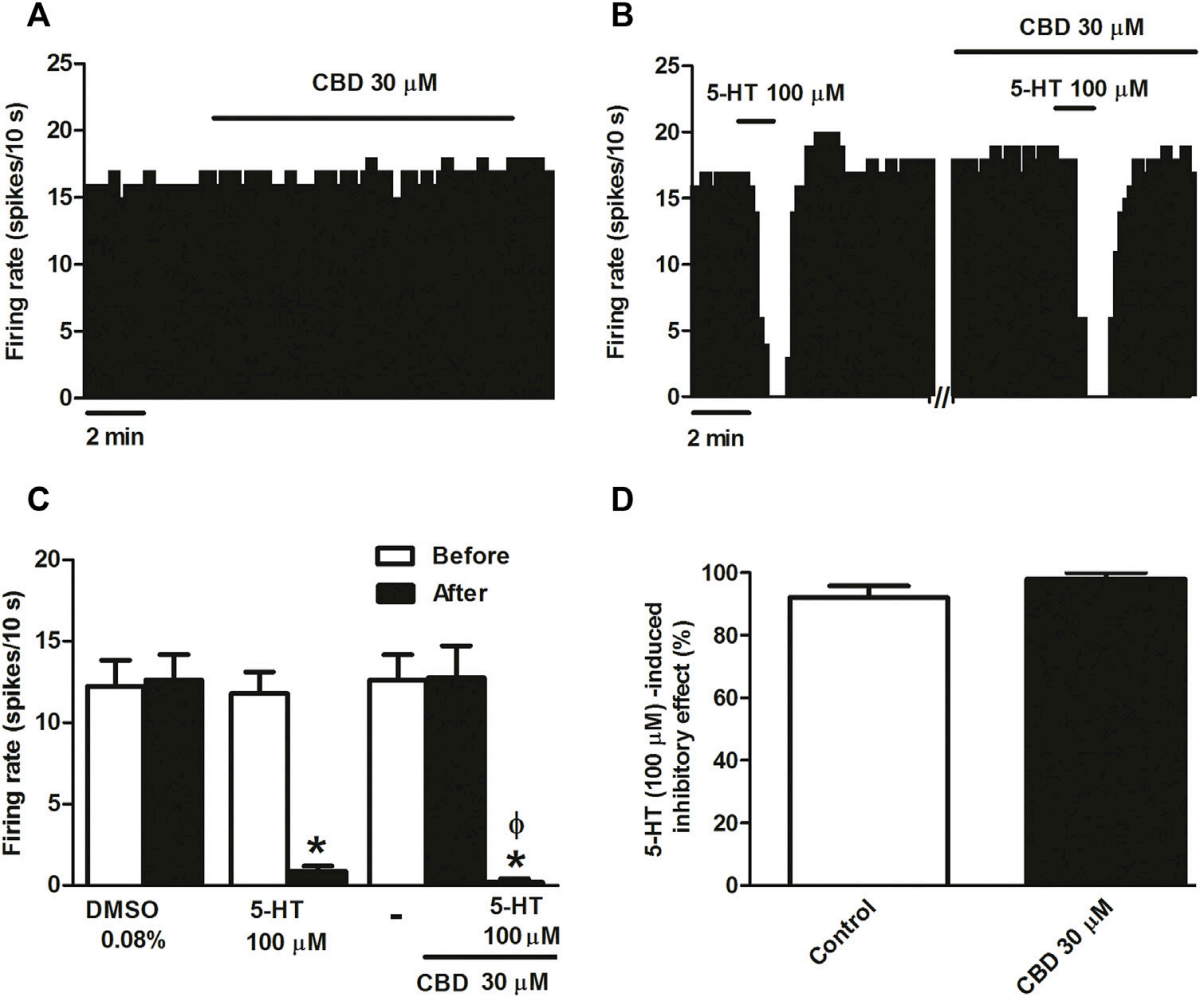
Effect of CBD on the firing rate of DRN 5-HT cells and on 5-HT-induced inhibition. **(A,B)** Representative examples of firing rate recordings from two DRN cells, which show the effect of CBD (30 μM) on the firing activity of 5-HT neurons **(A)** and the effect of CBD (30 μM) on 5-HT (100 μM)-induced inhibition **(B)**. Vertical lines refer to the integrated firing rate values (spikes per 10 s) and the horizontal lines represent the time scale. Drugs were perfused at the concentration and for the time indicated by the horizontal bars. ACSF was applied in the continuous presence of the vehicle (DMSO 0.08%). **(C)** Bar histograms showing the firing rate of 5-HT cells (mean ± SEM) before and after perfusion with the vehicle (DMSO 0.08%, *n* = 5), 5-HT (100 μM) (*n* = 5), CBD (30 μM) (*n* = 5), and CBD (30 μM) + 5-HT (100 μM) (*n* = 5). **(D)** Bar histograms showing the percentage of 5-HT (100 µM)-induced inhibition from the basal firing rate (mean ± SEM) in the absence and presence of CBD (30 μM) (*n* = 5). *p < 0.005 compared with the firing rate before drug application by paired Student’s t-test. *ΦP* < 0.005 compared with the firing rate after administration of CBD (30 μM) in the absence of 5-HT by paired Student’s t-test.

Several behavioral effects produced by CBD have been shown to be mediated by activation of the 5-HT_1A_ receptor. Therefore, we first tested the effect of CBD on DRN 5-HT cells, which express somatodendritic 5-HT_1A_ receptors. The vehicle (DMSO 0.08%) in which CBD was dissolved failed to affect the firing rate of DRN 5-HT cells (*n* = 5 Figure 1C). Similarly, perfusion with CBD (30 μM, 10 min) did not significantly change the firing rate of DRN 5-HT cells (maximal change in the firing rate: 0.56 ± 3.31%, *n* = 5) (Figures 1A,C), which suggests that the cannabinoid does not behave as an agonist of 5-HT_1A_ receptor located at the somatodendritic site of DRN 5-HT cells. To determine whether CBD regulates 5-HT_1A_ receptor function in the DRN, we tested the action of the cannabinoid on the inhibition induced by the endogenous 5-HT receptor agonist 5-HT. Administration of 5-HT (50–100 μM, 1 min) significantly reduced the firing rate of DRN 5-HT cells (maximal change in the firing rate: 92.09 ± 3.71%, *n* = 5 *p* < 0.005) (Figures 1B–D). However, CBD (30 μM, 10 min) did not significantly change the inhibition of DRN 5-HT cells produced by 5-HT (100 μM, 1 min) (maximal change in the firing rate in the presence of CBD: 98.00 ± 2.00%, n = 5) (Figures 1B–D). Thus, in the absence or presence of CBD (30 μM, 10 min), the firing rate after perfusion with 5-HT (100 μM, 1 min) was significantly lower than that before administration of the 5-HT_1A_ receptor agonist (0.20 ± 0.20 vs*.* 12.73 ± 2.00, *n* = 5, *p* < 0.005) (Figure 1C).

### Effect of CBD on the selective 5-HT_1A_ receptor agonist-induced inhibition of the firing activity of DRN 5-HT cells

**FIGURE 2:**
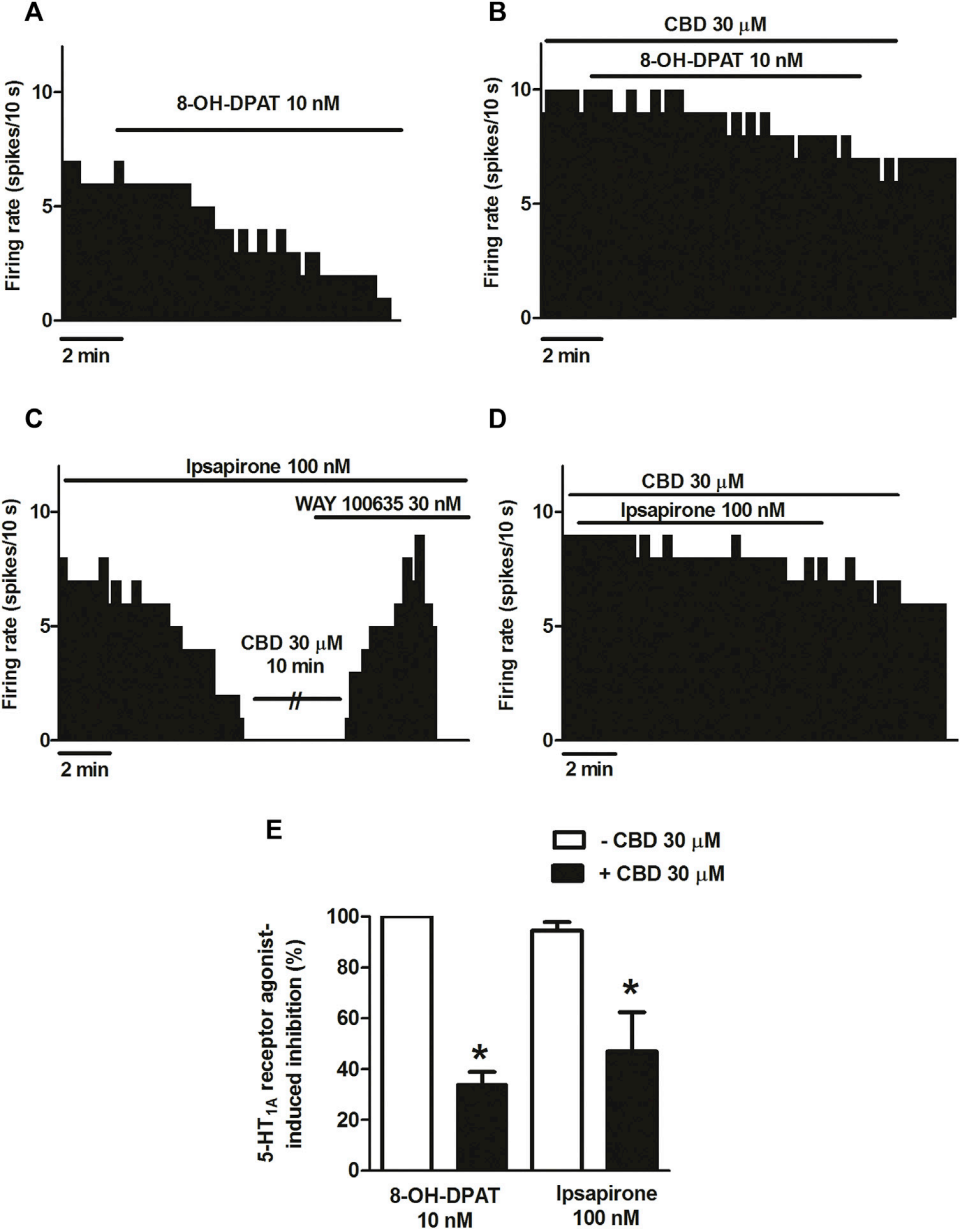
Effect of CBD on the selective 5-HT_1A_ receptor agonist-induced inhibition of the firing activity of DRN 5-HT cells. **(A–D)** Representative examples of firing rate recordings from DRN cells, which show the inhibition of the firing activity of 5-HT neurons by 8-OH-DPAT (10 nM) **(A)**, the blockade of 8-OH-DPAT (10 nM)-induced inhibition by CBD **(B)**, the inhibition of the firing rate of DRN 5-HT cells by ipsapirone (100 nM) and the effect of CBD (30 μM) or the 5-HT_1A_ receptor antagonist WAY100635 (30 nM) on that inhibition **(C)**, and the blockade of the effect of CBD on the inhibition of the firing activity of DRN 5-HT neurons induced by ipsapirone (100 nM) **(D)**. Vertical lines refer to the integrated firing rate values (spikes per 10 s), and the horizontal lines represent the time scale. Drugs were perfused at the concentration and for the time indicated by the horizontal bars. **(E)** Bar histograms showing the percentage of inhibition from the basal firing rate (mean ± SEM) induced by 8-OH-DPAT (10 nM) and ipsapirone (100 nM) in the absence (*n* = 5 and *n* = 13, respectively) and presence of CBD (30 μM, n = 6 and *n* = 10, respectively). *p < 0.05 compared with the inhibition induced by 8-OH-DPAT (10 nM) or ipsapirone (100 nM) in the absence of CBD (30 μM) by unpaired Student’s t-test.

According to described results, CBD failed to modify the inhibitory effect of 5-HT on the firing activity of DRN cells. It is known that 5-HT activates 5-HT receptors other than 5-HT_1A_. In order to avoid the putative action of 5-HT on non-5-HT_1A_ receptors, we used the more selective 5-HT_1A_ receptor agonist 8-OH-DPAT and ipsapirone. As expected, perfusion with 8-OH-DPAT (10 nM, 10 min) completely inhibited the firing rate of all recorded DRN 5-HT cells (Figures 2A,E) (*n* = 5). However, in the presence of CBD (30 μM), 8-OH-DPAT (10 nM) only partially inhibited DRN 5-HT cells (maximal change in the firing rate: 33.98 ± 4.93%, *n* = 6) (Figures 2B,E). Hence, in the presence of CBD, the inhibition induced by 8-OH-DPAT (10 nM) was reduced by 66% (*p* < 0.05, *n* = 6) (Figures 2B,E). To study whether the effect of CBD on 8-OH-DPAT-induced inhibition resembled that of another 5-HT_1A_ receptor agonist, we used the clinically studied piperizine and ipsapirone (100 nM), which has been shown to be more selective than buspirone for 5-HT_1A_ receptor. Perfusion with ipsapirone (100 nM, 10 min) inhibited the firing rate of DRN 5-HT cells (maximal change in the firing rate: 94.49 ± 3.34%, *n* = 13) (Figures 2C,E). Thus, the firing rate before ipsapirone perfusion was significantly higher than that after application of the 5-HT_1A_ receptor agonist (0.85 ± 0.13 Hz vs*.* 0.04 ± 0.02 Hz; *p* < 0.005, *n* = 13). As previously observed with 8-OH-DPAT (10 nM), in the presence of CBD, (30 μM) ipsapirone (100 nM)-induced inhibition of DRN 5-HT cells was decreased by 53%. Hence, the maximal inhibition in the firing rate of DRN 5-HT cells produced by ipsapirone (100 nM) in the presence of CBD was 47 ± 15.35% (*p* < 0.05, *n* = 10) (Figures 2D,E). With the purpose of studying if CBD (30 μM) behaved as a 5-HT_1A_ receptor antagonist to block ipsapirone-induced inhibition of DRN 5-HT cells, we tested its ability to restore the firing activity of 5-HT cells that had been completely inhibited by ipsapirone (100 nM). In these assays, CBD failed to reverse ipsapirone-induced inhibition, whereas perfusion with the 5-HT_1A_ receptor antagonist WAY100635 (30 nM) completely restored the firing activity of 5-HT cells by 97.05 ± 14.63% (basal firing rate: 0.80 ± 0.20 Hz; firing rate after WAY100635: 0.69 ± 0.09 Hz (*n* = 5) (Figure 2C).

### Mechanisms involved in the blockade by CBD of the ipsapirone-induced inhibitory effect on DRN 5-HT cells

**FIGURE 3:**
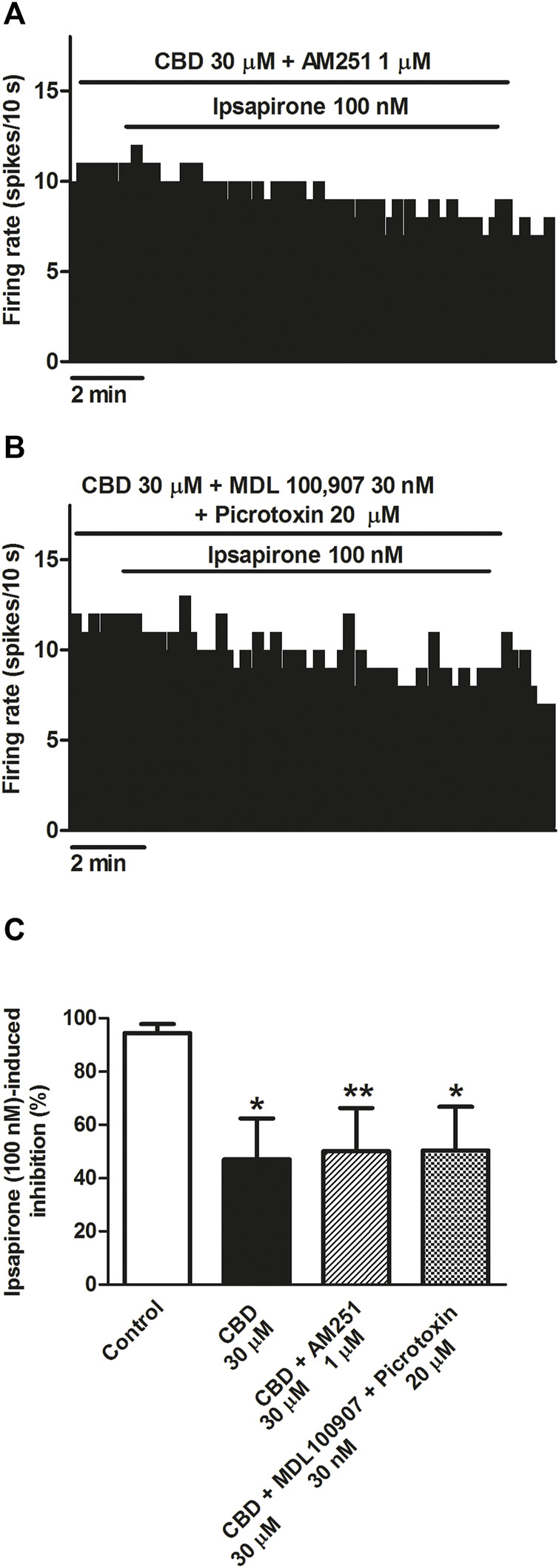
Mechanisms involved in the blockade by CBD of the ipsapirone-induced inhibitory effect on DRN 5-HT cells. **(A,B)** Representative examples of firing rate recordings from two DRN 5-HT neurons, which represent the effect of CBD on the ipsapirone (100 nM)-induced inhibition in the presence of the CB_1_ receptor antagonist AM251 (1 μM) **(A)** or in the presence of the GABA_A_ receptor antagonist picrotoxin (20 μM) and the 5-HT_2A_ receptor antagonist MDL100907 (30 nM) **(B)**. **(C)** Bar histograms showing the percentage of inhibition from the basal firing rate (mean ± SEM) induced by ipsapirone (100 nM) in the absence (*n* = 13) and presence of CBD (30 μM, *n* = 10), CBD (30 μM) + AM251 (1 µM) (*n* = 9) and CBD (30 μM) + MDL100907 (30 nM) + picrotoxin (20 μM) (n = 9). *p < 0.05, **p < 0.01 compared with ipsapirone (100 nM) group (control) by unpaired Student’s t-test.

CBD has been shown to target the 5-HT_2A_ receptor, CB_1_ receptor, or the GABA_A_ receptor, which are all present in the DRN and could modulate the firing activity of 5-HT cells. Therefore, we perfused CBD in the presence of the CB_1_ receptor antagonist AM251, the 5-HT_2A_ receptor antagonist MDL100907, or the GABA_A_ receptor antagonist picrotoxin, and we studied the inhibitory effect of ipsapirone (100 nM) on the firing activity of 5-HT cells. AM251 (1 µM) did not change the basal firing rate of the recorded 5-HT cells (FR before AM251: 1.2 ± 0.3 Hz vs*.* FR after AM251: 1.1 ± 0.3 Hz, n = 9) or the inhibitory effect of ipsapirone (effect in the presence of AM251: 100%, *n* = 2). In the presence of AM251 (1 µM), the blockade of ipsapirone-induced inhibition produced by CBD was not different from that in the absence of AM251 (49% vs*.* 53%, n = 9). Thus, the inhibition of the firing rate of DRN 5-HT cells produced by ipsapirone during perfusion with CBD and AM251 was 51.34 ± 15.74% (Figures 3A,C). Similarly, MDL100907 (30 nM) and picrotoxin (20 μM) failed to alter the firing activity of 5-HT cells (FR before MDL + picrotoxin: 0.8 ± 0.1 Hz vs*.* FR after MDL + picrotoxin: 0.9 ± 0.1 Hz, *n* = 9) or the inhibitory effect of ipsapirone (effect in the presence of MDL + picrotoxin: 100%, *n* = 2). Administration of MDL100907 (30 nM) and picrotoxin (20 μM) did not change the blockade produced by CBD (30 μM) on ipsapirone-induced (100 nM) inhibition (48% vs*.* 53%, *n* = 9). Hence, the inhibitory effect of ipsapirone (100 nM) on the firing rate of DRN 5-HT cells in the presence of CBD, MDL100907, and picrotoxin was 51.85 ± 15.87% (*n* = 9) (Figures 3B,C), which did not significantly differ from that in the absence of the 5-HT_2A_ and the GABA_A_ receptor antagonists. These results suggest that the CB_1_ receptor, the 5-HT_2A_ receptor, and the GABA_A_ receptor do not mediate the effect of CBD on the 5-HT_1A_ receptor agonist-induced inhibition of DRN 5-HT cells.

### Effect of ipsapirone and CBD on KCl-evoked calcium influx in DRN

**FIGURE 4:**
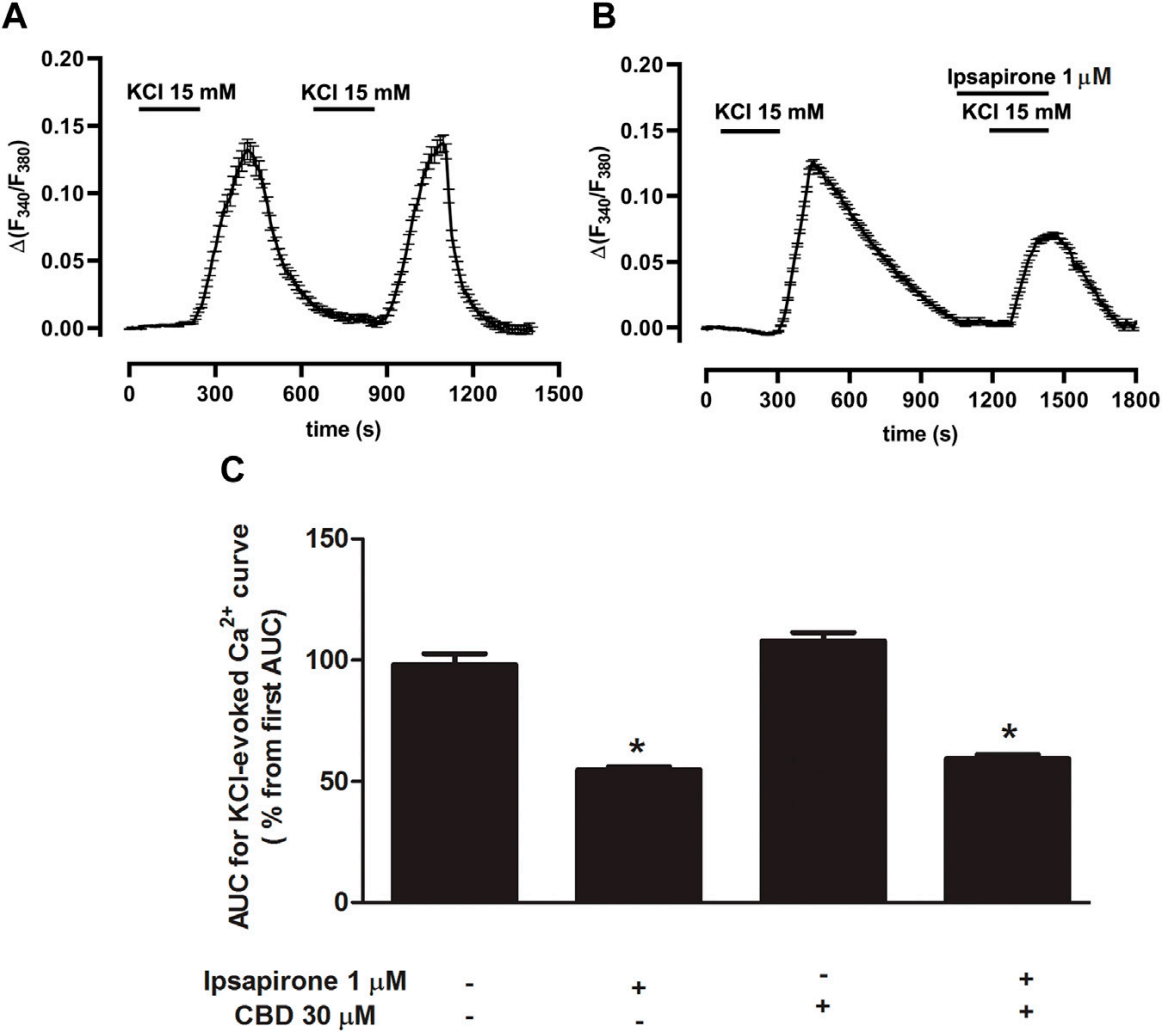
Effect of ipsapirone and CBD on KCl-evoked calcium influx in DRN. **(A,B)** Representative recordings of KCl-evoked (15 mM, 4 min) [Ca^2+^]i in DRN 5-HT cells in the absence **(A)** or presence of ipsapirone (1 µM) **(B)**. Vertical lines refer to the change in KCl-evoked 340/380 ratio, and the horizontal lines represent the time scale. **(C)** Bar histograms showing mean ± SEM of the AUC for KCl (15 mM)-evoked [Ca^2+^]i in DRN 5-HT cells in the absence (*n* = 29 cells, three slices from three rats) and presence of ipsapirone (1 µM) (*n* = 26 cells, three slices from three rats), CBD (30 μM, *n* = 21 cells, three slices from three rats), and CBD (30 µM) + ipsapirone (1 µM) (*n* = 17 cells, three slices from two rats). Each histogram represents the mean ± SEM of the percentage from the first AUC for KCl-evoked [Ca^2+^]i. *p < 0.001 compared with the corresponding groups without ipsapirone (1 µM) by two-way ANOVA (with CBD and ipsapirone as the main factors) followed by Bonferroni´s post-hoc test.

To study whether CBD modified 5-HT_1A_ receptor-mediated effects on [Ca^2+^]i in slices from the DRN, we first determined the increase in [Ca^2+^]i produced by a high concentration of KCl (15 mM) and then studied the effect of ipsapirone (1 μM). The area under the curve (AUC) for the KCl-evoked intracellular [Ca^2+^] peak was 31.4 ± 1.2 (*n* = 29 cells, three slices from three rats). A second stimulus with KCl increased [Ca^2+^]i to 29.8 ± 1.0 (Figure 4A). Thus, the percentage of the AUC for KCl-evoked [Ca^2+^]i with respect to the first AUC in DRN cells was 98.1 ± 4.6% (*n* = 29) (Figure 4C). When KCl-evoked [Ca^2+^]i was compared under different experimental conditions (ipsapirone/no ipsapirone and CBD/no CBD), significant differences were overall detected by two-way ANOVA for the main factors ipsapirone (F [1, 89] = 170.59; *p* < 0.0001) and CBD (F [1, 89] = 4.23; *p* = 0.04), although the CBD × ipsapirone interaction was not significant (F [1, 89] = 0.55). The Bonferroni´s post-hoc test only identified significant differences between ipsapirone/no ipsapirone groups, but not between CBD/no CBD groups. Thus, administration of ipsapirone (1 μM, 4 min) decreased KCl-evoked [Ca^2+^] peak to 54.8 ± 1.3% (*n* = 26 cells, three slices from three rats; *p* < 0.005 vs*.* control group) (Figures 4B,C), suggesting a blockade of VGCCs by 5-HT_1A_ receptor activation. Perfusion with CBD (30 μM) produced an increase in the KCl-evoked [Ca^2+^] curve in DRN 5-HT cells (98.1 ± 4.6% vs*.* 107.9 ± 3.5%, *n* = 21 cells, three slices from three rats; non-significant compared to the corresponding “no CBD” groups) (Figure 4C). Perfusion with ipsapirone (1 μM, 4 min) in the presence of CBD (30 μM, 4 min) decreased the KCl-evoked intracellular [Ca^2+^] peak to 59.4 ± 1.7% (*n* = 17 cells, three slices from two rats; *p* < 0.005 vs*.* CBD group) (Figure 4C). The effect of ipsapirone in the presence of CBD (30 μM) was not different from the effect in the absence of CBD (55% vs*.* 59%) (Figure 4C), which was in agreement with the lack of interaction between ipsapirone and CBD factors found by the two-way ANOVA (see above). Together, these data suggest that the inhibitory effect of ipsapirone on the KCl-evoked intracellular [Ca^2+^] peak is not altered by the previous perfusion with CBD.

## Discussion

The present work was undertaken to functionally characterize the effect of CBD on DRN 5-HT cells and 5-HT_1A_ autoreceptors. This is the first study in which the effect of the cannabinoid on neuronal excitability of 5-HT cells was explored *in vitro*. Our results reveal that CBD fails to affect the firing rate of DRN 5-HT cells or the effect of the endogenous neurotransmitter 5-HT, but it blocks the inhibitory effect of the selective 5-HT_1A_ receptor agonist´s 8-OH-DPAT and ipsapirone. The blockade of the inhibitory effect of ipsapirone is not mediated by the CB_1_, 5-HT_2A,_ or GABA_A_ receptors. We also concluded that CBD does not reverse the inhibitory effect of ipsapirone on the firing rate of DRN 5-HT cells. Finally, our data show that CBD fails to change the inhibitory effect of ipsapirone on the KCl-evoked [Ca^2+^]i peak. In this study, single-unit extracellular electrophysiological techniques and a calcium imaging approach were used in brain slice preparations, which show the advantage of preserving neuronal circuitry in comparison to other systems (i.e., cell culture) and isolating the responses mediated by somatodendritic 5-HT_1A_ receptors. CBD was applied at a single concentration, which was within the range of µM concentrations (10–30 µM) previously used to characterize the effect of cannabinoids on brain slice preparations (Mendiguren and Pineda, 2004; Mendiguren and Pineda, 2009; Castillo et al., 2010; Jones et al., 2010; Iannotti et al., 2014). According to kinetics models (Kenakin et al., 1997), this range of concentrations guarantees the access of lipophilic drugs to the receptor compartment when thick slices are used (500–600 μm; Castillo et al., 2010) Furthermore, the 5-HT_1A_ receptor agonists administrated in our study have been shown to activate GIRKs and inhibit VGCCs *via* 5-HT_1A_ receptor in the raphe nuclei at similar concentrations herein used, leading to reduction of the firing activity of 5-HT neurons and [Ca^2+^]i (Bayliss et al., 1997; Mendiguren and Pineda, 2009). We depolarized DRN 5-HT neurons with KCl to evoke [Ca^2+^]i peak through activation of VGCCs as was previously reported *in vitro* (Cheng et al., 1998).

In our study, CBD fails to change the neuronal activity of DRN 5-HT cells, but it negatively modulates the effect of 5-HT_1A_ receptor agonists on the firing rate of DRN 5-HT cells. These data demonstrate that CBD does not behave as an agonist at somatodendritic DRN 5-HT_1A_ autoreceptors regulating 5-HT neurons. The nature of the interaction of CBD with the 5-HT_1A_ receptor has been an issue of controversy. On the one hand, CBD has been reported to bind to the orthosteric site of the 5-HT_1A_ receptor and exert a modest agonistic effect. Thus, CBD displaced [3H]8-OH-DPAT, increased [35 S] GTPγS binding, and reduced cAMP in CHO cells, suggesting a direct partial agonism on the 5-HT_1A_ receptor (Russo et al., 2005). In view of these results, an inhibitory effect of CBD on the firing activity of 5-HT cells would have been expected in the DRN *in vitro.* However, in slices, the 5-HT_1A_ receptor is expressed at physiological levels, while in cell cultures, the 5-HT_1A_ receptor may be overexpressed, which could account for the differences observed. On the other hand, in line with our results, other authors have described an indirect action of the cannabinoid onto the serotonergic receptor by showing that CBD itself failed to stimulate [(35)S]GTPγS but changed 5-HT_1A_ receptor-mediated responses (Rock et al., 2012). In addition, several *in vivo* studies have attributed to CBD agonistic properties to the 5-HT_1A_ receptor because systemic administration of the cannabinoid reduces, via the 5-HT_1A_ receptor, the firing activity of DRN 5-HT cells (De Gregorio et al., 2019) and produces analgesic (De Gregorio et al., 2019; Jesus et al., 2019), antidepressant (Zanelati et al., 2010; Linge et al., 2016; Sartim et al., 2016) or anxiolytic effects (Resstel et al., 2009). However, *in vivo* studies measure the serotonergic activity in the whole animal and consequently do not distinguish between the action of CBD on DRN 5-HT_1A_ autoreceptors from that on 5-HT_1A_ receptors located in different brain areas (i.e., hippocampus). Therefore, our results in the DRN would not be directly comparable to the data *in vivo* where the activation of 5-HT_1A_ receptors at hippocampal pyramidal neurons could contribute to indirectly exerting negative feedback on DRN 5-HT cells. There is only one electrophysiological evidence in brain hippocampal slices comparable to our methodological procedure in which CBD reduced the frequency of spontaneous discharge by acting as an agonist at the 5-HT_1A_ receptor (Ledgerwood et al., 2011). Yet, it is important to note that some agonists at the hippocampal postsynaptic 5-HT_1A_ receptor could negatively regulate 5-HT_1A_ autoreceptors in the DRN.

Our study performed in slices from the DRN indicates that CBD does not behave as a competitive antagonist at the orthosteric site of the 5-HT_1A_ receptor since it does not reverse the effect of ipsapirone. In line with this, previous radioligand binding assays in rat brainstem membranes demonstrated that CBD fails to displace [^3^H]-8-OH-DPAT from the specific 5-HT_1A_ receptor binding site, although it enhances the ability of 8-OH-DPAT to stimulate [35) S]GTPγS binding (Rock et al., 2012). Our data show that CBD blocks, rather than enhancing, the inhibitory effects of 8-OH-DPAT and ipsapirone on the firing rate but not that of the endogenous neurotransmitter 5-HT. The discrepancy between the effect of CBD on 8-OH-DPAT observed in binding assays and electrophysiological studies could arise from the differences in the preparation used (brainstem membranes vs*.* slices) and/or the concentration of the cannabinoid tested (100 nM vs*.* 30 µM). Thus, GTPγS binding experiments were performed in rat brainstem membranes, which contain different brain areas and cell types that may contribute to the overall effect of CBD on 8-OH-DPAT. In contrast, our experiments were carried out in slices from the DRN tissue in which the measured effects correspond specifically to neurons. Additionally, in binding assays, the concentration of drugs is not equivalent to that in electrophysiological studies due to differences in the experimental conditions to promote binding to the receptor. Finally, there may be functionally selective regulation of 5-HT_1A_ receptors that could explain why two responses coupled to the same receptor may be differentially affected.

With regard to the lack of effect of CBD on the 5-HT-induced inhibition, it is well known that the 5-HT_1A_ receptor agonists used display higher selectivity for 5-HT_1A_ receptors than 5-HT. Hence, 5-HT activates 5-HT_7_ receptors (Roberts et al., 2004; Albert and Vahid-Ansari, 2019), which regulate GABAergic neurons. 5-HT also activates the presynaptic 5-HT_1B_ receptors (Morikawa et al., 2000; Adell et al., 2001), some of which are heteroreceptors inhibiting glutamatergic neurotransmission in the DRN (Lemos et al., 2006; Geddes et al., 2016). Therefore, the lack of effect of CBD on 5-HT-induced inhibition may be explained by the activation of non-5-HT_1A_ receptors that mask the 5-HT_1A_ receptor-mediated effect. On the other hand, several further studies indicate that 5-HT and selective 5-HT_1A_ receptor agonists may bind and activate the 5-HT_1A_ receptor differently. Thus, there are neurons that fail to respond to medium–high doses of 5-HT (50–100 μM) but are completely inhibited by 8-OH-DPAT or ipsapirone. Moreover, differences in coupling to adenylate cyclase have been described after activation of the 5-HT_1A_ receptor by 5-HT or 8-OH-DPAT (Varrault and Bockaert, 1992; Abigail and Xiaohua, 2010).

Integrating electrophysiological data, a proposal for the mechanism of action of CBD on the presynaptic DRN 5-HT_1A_ receptor would be a negative allosteric modulation of the receptor or its binding to Gi/o protein and/or GIRK. One hypothesis is that this modulation can only be seen with the selective 5-HT_1A_ receptor agonists but not with 5-HT due to CBD signaling bias at the DRN 5-HT_1A_ receptor.

Our results show that ipsapirone reduces KCl-evoked [Ca^2+^]i peak probably through inhibition of VGCCs as was previously shown with other 5-HT_1A_ receptor agonists in the raphe and other brain nuclei (Penington and Kelly, 1990; Bayliss et al., 1997; Cheng et al., 1998). However, in contrast to electrophysiological assays, CBD does not modify the inhibitory effect of ipsapirone on KCl-evoked [Ca^2+^]i. These data would further support the functional selectivity of CBD at the 5-HT_1A_ receptor to differently regulate specific Gi/o protein-coupled intracellular signaling pathways. In the DRN, 5-HT_1A_ autoreceptor agonists activate GIRKs and inhibit VGCCs, but it has been shown that the main effect on the activity of 5-HT cells results from the stimulation of GIRKs *via* G protein βγ subunit (Courtney and Ford, 2016; Albert and Vahid-Ansari, 2019). Therefore, one could speculate that CBD would preferentially regulate 5-HT_1A_ receptor-dependent activation of GIRKS and subsequently the inhibitory effect of ipsapirone on the firing rate rather than VGCC-mediated decrease in [Ca^2+^]i.

According to our data, the effect of CBD on the inhibition produced by the selective 5-HT_1A_ receptor agonists occurs through an indirect mechanism. Recently, a variety of molecular targets have been described for CBD (De Almeida and Devi, 2020; Britch et al., 2021). Among them, the CB_1_, 5-HT_2A,_ and GABA_A_ receptors could contribute to the modulation of the firing rate of 5-HT cells in rat brain slices from the DRN as was previously reported in functional studies (Liu et al., 2000; Mendiguren and Pineda, 2009). Furthermore, CB_1_ mRNA is present in a significant number of non-5-HT cells (probably GABAergic interneurons) in the DRN (Häring et al., 2007) and 5-HT_2A_ receptors modulate the local GABAergic neurons, which project to 5-HT cells (Liu et al., 2000). However, perfusion with selective CB_1_, HT_2A,_ and the GABA_A_ receptor antagonists does not change the effect of CBD on 8-OH-DPAT and ipsapirone-induced inhibition, which rules out the involvement of these receptors. CB_1_ receptors are also found in glutamatergic afferents from the mPFC to the nucleus (Marsicano and Lutz, 1999). Cannabinoids regulate mPFC inputs onto both 5-HT and GABAergic neurons, but it has been shown that glutamatergic synapses onto GABA neurons are more sensitive to CB_1_ receptor-mediated inhibition in the DRN (Geddes et al., 2016). Therefore, CB_1_ receptor activation in glutamatergic neuron terminals would lead to reduction of the inhibitory GABAergic input to 5-HT cells. If CBD had activated CB_1_ receptors located in the mPFC, then the CB_1_ receptor antagonist AM251 and/or the GABA_A_ receptor antagonist picrotoxin would have altered the effect of CBD on 5-HT_1A_ receptor-mediated inhibition, which was not the case. It is important to note that although glutamatergic terminals from mPFC may be externally activated in DRN slices (Geddes et al., 2016), under spontaneous conditions in brain slices, they do not appear to be active.

According to our study, we cannot rule out the involvement of the putative CB_2_ receptor in the effect of CBD at the concentration used, in view of the evidence showing its localization in the rat brainstem (Gong et al., 2006; Onaivi et al., 2006) and the fact that CBD behaves as a partial agonist at CB_2_ receptor (Shahbazi et al., 2020). However, to date, results from molecular and functional studies do not reveal the presence of CB_2_ receptor modulating DRN 5-HT cells. In conclusion, CBD does not activate somatodendritic 5-HT_1A_ autoreceptors, but it hindered the inhibitory effect produced by two different selective 5-HT_1A_ receptor agonists by a mechanism that does not involve CB_1_, 5-HT_2A,_ or GABA_A_ receptors. Our data support a negative allosteric modulation of DRN somatodendritic 5-HT_1A_ receptor by CBD. This study could help better understand the diverse effects of CBD on 5-HT_1A_ receptors in the brain and its possible involvement in the pharmacological effects of the non-psychoactive cannabinoid. Functional characterization of the effect of CBD on DRN 5-HT could be of interest in view of the contribution of this nucleus and 5-HT_1A_ autoreceptors to the regulation of the emotional state, anxiety, and arousal or control of pain, functions that are influenced by cannabinoids (Lowry et al., 2008; McDevitt and Neumaier, 2011; Campion et al., 2016) The use of brain slices has allowed us to study the somatodendritic regulation of 5-HT neurons isolated from external influences. However, in the whole animal, these results should be added to further regulation arising from long inputs or other local circuits active *in vivo* (such as GABAergic or glutamatergic), which could affect 5-HT cells. In addition, the technical approach used in this study makes it difficult to elucidate the exact mechanism by which CBD modulates 5-HT_1A_ receptors in the DRN *in vitro*.

## Data Availability

The raw data supporting the conclusion of this article will be made available by the authors, without undue reservation.

## Abbreviations

ACSF, artificial cerebrospinal fluid; AM251, N-(piperidin-1-yl)-5-(4-iodophenyl)-1-(2,4-dichlorophenyl)-4-methyl-1H-pyrazole-3-carboxamide; CBD, cannabidiol; DMSO, dimethylsulfoxide; DRN, dorsal raphe nucleus; GIRKs, G protein–coupled inwardly rectifying potassium channels; 5-HT, serotonin; 8-OH-DPAT (±)-8-hydroxy-2-(di-n-propylamino) tetralin hydrobromide; GABA, *γ*-aminobutyric acid; MDL100907, R-(+)-α-(2,3-dimethoxyphenyl)-1-[2-(4-fluorophenyl)ethyl]-4-piperinemethanol; PE, phenylephrine; ∆^9^-THC, ∆^9^-tetrahydrocannabinol; VGCCs, voltage-gated Ca^+2^ channels; WAY100635, N-[2-[4-(2-methoxyphenyl)-1-piperazinyl]ethyl]-N-2-yridinylcyclohexanecarboxamide.

## References

[B1] Abigail M. Xiaohua Li. (2010). 5-HT_1A_ receptor-regulated signal transduction pathways in brain. Cell. Signal. 22 (10), 1406–1412. 10.1016/j.cellsig.2010.03.019 20363322 PMC2903656

[B2] Adell A. Celada P. Artigas F. (2001). The role of 5-HT_1B_ receptors in the regulation of serotonin cell firing and release in the rat brain. J. Neurochem. 79, 172–182. 10.1046/j.1471-4159.2001.00550.x 11595769

[B3] Aghajanian G. K. Lakoski J. M. (1984). Hyperpolarization of serotonergic neurons by serotonin and LSD: Studies in brain slices showing increased K^+^ conductance. Brain Res. 305, 181–185. 10.1016/0006-8993(84)91137-5 6331598

[B4] Albert P. R. Vahid-Ansari F. (2019). The 5-HT_1A_ receptor: Signaling to behavior. Biochimie 161, 34–45. 10.1016/j.biochi.2018.10.015 31079617

[B5] Andrade R. Huereca D. Lyons J. G. Andrad e. E. M. McGregor K. M. (2015). 5-HT_1A_ receptor-mediated Autoinhibition and the control of serotonergic cell firing. ACS Chem. Neurosci. 6 (7), 1110–1115. 10.1021/acschemneuro.5b00034 25913021 PMC4849862

[B6] Bakas T. Van Nieuwenhuijzen P. S. Devenish S. O. McGregor I. S. Arnold J. C. Chebib M. (2017). The direct actions of cannabidiol and 2-arachidonoyl glycerol at GABA A receptors. Pharmacol. Res. 119, 358–370. 10.1016/j.phrs.2017.02.022 28249817

[B7] Bambico F. R. Katz N. Debonnel G. Gobbi G. (2007). Cannabinoids elicit antidepressant-like behavior and activate serotonergic neurons through the medial prefrontal cortex. J. Neurosci. 27 (43), 11700–11711. 10.1523/JNEUROSCI.1636-07.2007 17959812 PMC6673235

[B8] Bayliss D. A. Li Y. W. Talley E. M. (1997). Effects of serotonin on caudal raphe neurons: Inhibition of N- and P/Q-type calcium channels and the afterhyperpolarization. J. Neurophysiol. 77 (3), 1362–1374. 10.1152/jn.1997.77.3.1362 9084603

[B9] Bolognini D. Rock E. M. Cluny N. L. Cascio M. G. Limebeer C. L. Duncan M. (2013). Cannabidiolic acid prevents vomiting in Suncus murinus and nausea-induced behavior in rats by enhancing 5-HT_1A_ receptor activation. Br. J. Pharmacol. 168 (6), 1456–1470. 10.1111/bph.12043 23121618 PMC3596650

[B10] Braida D. Limonta V. Malabarba L. Zani A. Sala M. (2007). 5-HT_1A_ receptors are involved in the anxiolytic effect of Delta9-tetrahydrocannabinol and AM 404, the anandamide transport inhibitor, in Sprague-Dawley rats. Eur. J. Pharmacol. 555 (2-3), 156–163. 10.1016/j.ejphar.2006.10.038 17116299

[B11] Britch S. C. Babalonis S. Walsh S. L. (2021). Cannabidiol: Pharmacology and therapeutic targets. Psychopharmacol. Berl. 238 (1), 9–28. 10.1007/s00213-020-05712-8 PMC7796924 33221931

[B12] Campion K. N. Saville K. A. Morgan M. M. (2016). Relative contribution of the dorsal raphe nucleus and ventrolateral periaqueductal gray to morphine antinociception and tolerance in the rat. Eur. J. Neurosci. 44 (9), 2667–2672. 10.1111/ejn.13378 27564986 PMC5300757

[B13] Castillo A. Tolón M. R. Fernández-Ruiz J. Romero J. Martinez-Orgado J. (2010). The neuroprotective effect of cannabidiol in an *in vitro* model of newborn hypoxic-ischemic brain damage in mice is mediated by CB(2) and adenosine receptors. Neurobiol. Dis. 37 (2), 434–440. 10.1016/j.nbd.2009.10.023 19900555

[B14] Celada P. Puig M. V. Casanovas J. M. Guillazo G. Artigas F. (2001). Control of dorsal raphe serotonergic neurons by the medial prefrontal cortex: Involvement of serotonin-1A, GABA(A), and glutamate receptors. J. Neurosci. 21 (24), 9917–9929. 10.1523/jneurosci.21-24-09917.2001 11739599 PMC6763042

[B15] Challis C. Boulden J. Veerakumar A. Espallergues J. Vassoler F. M. Christopher Pierce R. (2013). Raphe GABAergic neurons mediate the acquisition of avoidance after social defeat. J. Neurosci. 33 (35), 13978–13988. 10.1523/JNEUROSCI.2383-13.2013 23986235 PMC3756748

[B16] Cheng L. L. Wang S. J. Gean P. W. (1998). Serotonin depresses excitatory synaptic transmission and depolarization-evoked Ca^2+^ influx in rat basolateral amygdala via 5-HT_1A_ receptors. Eur. J. Neurosci. 10 (6), 2163–2172. 10.1046/j.1460-9568.1998.00229.x 9753102

[B17] Courtney A. N. Ford C. P. (2016). Mechanisms of 5-HT_1A_ receptor-mediated transmission in dorsal raphe serotonin neurons. J. Physiol. 594 (4), 953–965. 10.1113/JP271716 26634643 PMC4753271

[B18] De Almeida D. L. Devi L. A. (2020). Diversity of molecular targets and signaling pathways for CBD. Pharmacol. Res. Perspect. 8 (6), e00682. 10.1002/prp2.682 33169541 PMC7652785

[B19] De Gregorio D. McLaughlin R. J. Posa L. Ochoa-Sanchez R. Enns J. Lopez-Canul M. (2019). Cannabidiol modulates serotonergic transmission and reverses both allodynia and anxiety-like behavior in a model of neuropathic pain. Pain 160 (1), 136–150. 10.1097/j.pain.0000000000001386 30157131 PMC6319597

[B20] Fernández-Ruiz J. Sagredo O. Pazos M. R. García C. Pertwee R. Mechoulam R. (2013). Cannabidiol for neurodegenerative disorders: Important new clinical applications for this phytocannabinoid? Br. J. Clin. Pharmacol. 75 (2), 323–333. 10.1111/j.1365-2125.2012.04341.x 22625422 PMC3579248

[B21] Garcia-Garcia L. A. Newman-Tancredi A. Leonardo E. D. (2014). 5-HT(1A) [corrected] receptors in mood and anxiety: Recent insights into autoreceptor versus heteroreceptor function. Psychopharmacol. Berl. 231 (4), 623–636. 10.1007/s00213-013-3389-x PMC3927969 24337875

[B22] Geddes S. D. Assadzada S. Lemelin D. Sokolovski A. Bergeron R. Haj-Dahmane S. (2016). Target-specific modulation of the descending prefrontal cortex inputs to the dorsal raphe nucleus by cannabinoids. Proc. Natl. Acad. Sci. U. S. A. 113 (19), 5429–5434. 10.1073/pnas.1522754113 27114535 PMC4868450

[B23] Gobbi G. Bambico F. R. Mangieri R. Bortolato M. Campolongo P. Solinas M. (2005). Antidepressant-like activity and modulation of brain monoaminergic transmission by blockade of anandamide hydrolysis. Proc. Natl. Acad. Sci. U. S. A. 102, 18620–18625. 10.1073/pnas.0509591102 16352709 PMC1317988

[B24] Gomes F. V. Del Bel E. A. Guimarães F. S. (2013). Cannabidiol attenuates catalepsy induced by distinct pharmacological mechanisms via 5-HT_1A_ receptor activation in mice Prog. Neuropsychopharmacol. Biol Psychiatry 46, 43–47. 10.1016/j.pnpbp.2013.06.005 23791616

[B25] Gong J. P. Onaivi E. S. Ishiguro H. Liu Q. R. Tagliaferro P. A. Brusco A. (2006). Cannabinoid CB2 receptors: Immunohistochemical localization in rat brain. Brain Res. 1071 (1), 10–23. 10.1016/j.brainres.2005.11.035 16472786

[B26] Grynkiewicz G. Poenie M. Tsien R. Y. (1985). A new generation of Ca2+ indicators with greatly improved fluorescence propertiesHajós, M., Sharp, TBurst-firing activity of presumed 5-HT neurones of the rat dorsal raphe nucleus: Electrophysiological analysis by antidromic stimulation. J. Biol. ChemBrain Res. 260740 (61-4), 162162–162508. 10.1016/s0006-8993(96)00869-4

[B62] Hajós M. Sharp T. (1996). Burst-firing activity of presumed 5-HT neurones of the rat dorsal raphe nucleus: electrophysiological analysis by antidromic stimulation. Brain Res. 740 (1-4), 162162–162508. 10.1016/j.brainres.2005.11.035 8973810

[B27] Häring M. Marsicano G. Lutz B. Monory K. (2007). Identification of the cannabinoid receptor type 1 in serotonergic cells of raphe nuclei inmice. Neuroscience 146, 1212–1219. 10.1016/j.neuroscience.2007.02.021 17383106

[B28] Hernández-Vázquez F. Garduño J. Hernández-López S. (2019). GABAergic modulation of serotonergic neurons in the dorsal raphe nucleus. Rev. Neurosci. 30 (3), 289–303. 10.1515/revneuro-2018-0014 30173207

[B29] Iannotti F. A. Hill C. L. Leo A. Alhusaini A. Soubrane C. Mazzarella E. (2014). Nonpsychotropic plant cannabinoids, cannabidivarin (CBDV) and cannabidiol (CBD), activate and desensitize transient receptor potential vanilloid 1 (TRPV1) channels *in vitro*: Potential for the treatment of neuronal hyperexcitability. ACS Chem. Neurosci. 5 (11), 1131–1141. 10.1021/cn5000524 25029033

[B30] Jesus C. H. A. Redivo D. D. B. Gasparin A. T. Sotomaior B. B. De Carvalho M. C. Genaro K. (2019). Cannabidiol attenuates mechanical allodynia in streptozotocin-induced diabetic rats via serotonergic system activation through 5-HT_1A_ receptors. Brain Res. 1715, 156–164. 10.1016/j.brainres.2019.03.014 30898678

[B31] Jones N. A. Hill A. J. Smith I. Bevan S. A. Williams C. M. Whalley B. J. (2010). Cannabidiol displays antiepileptiform and antiseizure properties *in vitro* and *in vivo* . J. Pharmacol. Exp. Ther. 332 (2), 569–577. 10.1124/jpet.109.159145 19906779 PMC2819831

[B32] Kenakin T. (1997). The Pharmacologic analysis of drug-receptor interaction. second edition. New York: Lippicontt-Raven press, 137–175.Concentrations of Drugs in the receptor compartment

[B33] Ledgerwood C. J. Greenwood S. M. Brett R. R. Pratt J. A. Bushell T. J. (2011). Cannabidiol inhibits synaptic transmission in rat hippocampal cultures and slices via multiple receptor pathways. Br. J. Pharmacol. 162, 286–294. 10.1111/j.1476-5381.2010.01015.x 20825410 PMC3012422

[B34] Lemos J. C. Pan Y. Z. Ma X. Lamy C. Akanwa A. C. Beck S. G. (2006). Selective 5-HT receptor inhibition of glutamatergic and GABAergic synaptic activity in the rat dorsal and median raphe. Eur. J. Neurosci. 24 (12), 3415–3430. 10.1111/j.1460-9568.2006.05222.x 17229091 PMC2837807

[B35] Linge R. Jiménez-Sánchez L. Campa L. Pilar-Cuéllar F. Vidal R. Pazos A. (2016). Cannabidiol induces rapid-acting antidepressant-like effects and enhances cortical 5-HT/glutamate neurotransmission: Role of 5-HT_1A_ receptors. Neuropharmacology 103, 16–26. 10.1016/j.neuropharm.2015.12.017 26711860

[B36] Liu R. Jolas T. Aghajanian G. (2000). Serotonin 5-HT(2) receptors activate local GABA inhibitory inputs to serotonergic neurons of the dorsal raphe nucleus. Brain Res. 873 (1), 34–45. 10.1016/s0006-8993(00)02468-9 10915808

[B37] Lowry C. A. Hale M. W. Evans A. K. Heerkens J. Staub D. R. Gasser P. J. (2008). Serotonergic systems, anxiety, and affective disorder: Focus on the dorsomedial part of the dorsal raphe nucleus. Ann. N. Y. Acad. Sci. 1148, 86–94. 10.1196/annals.1410.004 19120094

[B38] Marinho A. L. Z. Vila-Verde C. Fogaça M. V. Guimarães F. S. (2015). Effects of intra-infralimbic prefrontal cortex injections of cannabidiol in the modulation of emotional behaviors in rats: Contribution of 5HT_1A_ receptors and stressful experiences. Behav. Brain Res. 286, 49–56. 10.1016/j.bbr.2015.02.023 25701682

[B39] Marsicano G. Lutz B. (1999). Expression of the cannabinoid receptor CB1 in distinct neuronal subpopulations in the adult mouse forebrain. Eur. J. Neurosci. 11 (12), 4213–4225. 10.1046/j.1460-9568.1999.00847.x 10594647

[B40] McDevitt R. A. Neumaier J. F. (2011). Regulation of dorsal raphe nucleus function by serotonin autoreceptors: A behavioral perspective. J. Chem. Neuroanat. 41 (4), 234–246. 10.1016/j.jchemneu.2011.05.001 21620956 PMC3137942

[B41] Mendiguren A. Pineda J. (2004). Cannabinoids enhance N-methyl-D-aspartate-induced excitation of locus coeruleus neurons by CB_1_ receptors in rat brain slices Neurosci. Neurosci. Lett. 363 (1), 1–5. 10.1016/j.neulet.2004.02.073 15157983

[B42] Mendiguren A. Aostri E. Pineda J. (2018). Regulation of noradrenergic and serotonergic systems by cannabinoids: Relevance to cannabinoid-induced effects. Life Sci. 192, 115–127. 10.1016/j.lfs.2017.11.029 29169951

[B43] Mendiguren A. Pineda J. (2009). Effect of the CB_1_ receptor antagonists rimonabant and AM 251 on the firing rate of dorsal raphe nucleus neurons in rat brain slices. Br. J. Pharmacol. 158 (6), 1579–1587. 10.1111/j.1476-5381.2009.00434.x 19845674 PMC2795224

[B59] Monti J. M. (2010). The structure of the dorsal raphe nucleus and its relevance to the regulation of sleep and wakefulness. Sleep Med. Rev. 14 (5), 307–317. 10.1016/j.smrv.2009.11.004 20153669

[B44] Morikawa H. Manzoni O. J. Crabbe J. C. Williams J. T. (2000). Regulation of central synaptic transmission by 5-HT1B auto-and-heteroreceptors. Mol. Pharmacol. 58, 1271–1278. 10.1124/mol.58.6.1271 11093763

[B45] Onaivi E. S. Ishiguro H. Gong J. P. Patel S. Perchuk A. Meozzi P. A. (2006). Discovery of the presence and functional expression of cannabinoid CB2 receptors in brain. Ann. N. Y. Acad. Sci. 1074, 514–536. 10.1196/annals.1369.052 17105950

[B46] Penington N. J. Kelly J. S. (1990). Serotonin receptor activation reduces calcium current in an acutely dissociated adult central neuron. Neuron 4 (5), 751–758. 10.1016/0896-6273(90)90201-p 2140514

[B47] Peyron C. Rampon C. Petit J. M. Luppi P. H. Piñeyro G. Blier P. (2018). Sub-regions of the dorsal raphé nucleus receive different inputs from the brainstem.Autoregulation of serotonin neurons: Role in antidepressant drug action. Sleep. Med.Pharmacol. Rev. 4951 (3), 53533–56391. 10.1016/j.sleep.2018.07.002

[B60] Piñeyro G. Blier P. (1999). Autoregulation of serotonin neurons: Role in antidepressant drug action. Pharmacol. Rev. 51 (3), 533–591. 10471417

[B48] Pisanti S. Malfitano A. M. Ciaglia E. Lamberti A. Ranieri R. Cuomo G. (2017). Cannabidiol: State of the art and new challenges for therapeutic applications. Pharmacol. Ther. 175, 133–150. 10.1016/j.pharmthera.2017.02.041 28232276

[B49] Resstel L. B. Tavares R. F. Lisboa S. F. Joca S. R. Corrêa F. M. Guimarães F. S. (2009). 5-HT_1A_ receptors are involved in the cannabidiol-induced attenuation of behavioural and cardiovascular responses to acute restraint stress in rats. Br. J. Pharmacol. 156 (1), 181–188. 10.1111/j.1476-5381.2008.00046.x 19133999 PMC2697769

[B50] Roberts C. Thomas D. R. Bate S. T. Kewa J. N. C. (2004). GABAergic modulation of 5-HT_7_ receptor-mediated effects on 5-HT efflux in the Guinea-pig dorsal raphe nucleus. Neuropharmacology 46, 935–941. 10.1016/j.neuropharm.2004.01.010 15081790

[B51] Rock E. M. Bolognini D. Limebeer C. L. Cascio M. G. Anavi-Goffer S. Fletcher P. J. (2012). Cannabidiol, a non-psychotropic component of cannabis, attenuates vomiting and nausea-like behavior via indirect agonism of 5-HT_1A_ somatodendritic autoreceptors in the dorsal raphe nucleus. Br. J. Pharmacol. 165 (8), 2620–2634. 10.1111/j.1476-5381.2011.01621.x 21827451 PMC3423241

[B52] Rodrigues da Silva N. Gomes Villela F. Sonego Buzolin A. Rodrigues da Silva N. Guimarães Silveira F. (2020). Cannabidiol attenuates behavioral changes in a rodent model of schizophrenia through 5-HT_1A_, but not CB_1_ and CB_2_ receptors. Pharmacol. Res. 156, 104749. 10.1016/j.phrs.2020.104749 32151683

[B53] Russo E. B. Burnett A. Hall B. Parker K. K. (2005). Agonistic properties of cannabidiol at 5-HT_1A_ receptors. Neurochem. Res. 30 (8), 1037–1043. 10.1007/s11064-005-6978-1 16258853

[B54] Sartim A. G. Guimarães F. S. Joca S. R. (2016). Antidepressant-like effect of cannabidiol injection into the ventral medial prefrontal cortex-Possible involvement of 5-HT_1A_ and CB_1_ receptors. Behav. Brain Res. 303, 218–227. 10.1016/j.bbr.2016.01.033 26801828

[B55] Shahbazi F. Grandi V. Banerjee A. Trant J. F. (2020). Cannabinoids and cannabinoid receptors: The story so far. iScience 23 (7), 101301. 10.1016/j.isci.2020.101301 32629422 PMC7339067

[B56] Silvestro S. Schepici G. Bramanti P. Mazzon E. (2020). Molecular targets of cannabidiol in experimental models of Neurological Disease. Molecules 25 (21), 5186. 10.3390/molecules25215186 PMC7664437 33171772

[B57] Varrault A. Bockaert J. (1992). Differential coupling of 5-HT_1A_ receptors occupied by 5-HT or 8-OH-DPAT to adenylyl cyclase. Naunyn. Schmiedeb. Arch. Pharmacol. 346 (4), 367–374. 10.1007/BF00171076 1436121

[B58] Zanelati T. V. Biojone C. Moreira F. A. Guimarães F. S. Joca S. R. (2010). Antidepressant-like effects of cannabidiol in mice: Possible involvement of 5-HT_1A_ receptors. Br. J. Pharmacol. 159 (1), 122–128. 10.1111/j.1476-5381.2009.00521.x 20002102 PMC2823358

